# Differential genetic correlations across major psychiatric disorders between Eastern and Western countries

**DOI:** 10.1111/pcn.13498

**Published:** 2022-11-17

**Authors:** Takeo Saito, Masashi Ikeda, Chikashi Terao, Takuma Ashizawa, Masami Miyata, Satoshi Tanaka, Tetsufumi Kanazawa, Tadafumi Kato, Taro Kishi, Nakao Iwata

**Affiliations:** ^1^ Department of Psychiatry Fujita Health University School of Medicine Toyoake Japan; ^2^ Laboratory for Statistical and Translational Genetics RIKEN Center for Integrative Medical Sciences Yokohama Japan; ^3^ Clinical Research Center Shizuoka General Hospital Shizuoka Japan; ^4^ The Department of Applied Genetics, The School of Pharmaceutical Sciences University of Shizuoka Shizuoka Japan; ^5^ Development Office for Genomic Medical Research Center, Research Promotion and Support Headquarters Fujita Health University Toyoake Japan; ^6^ National Hospital Organization Higashi Owari Hospital Nagoya Japan; ^7^ Clinical Research Center National Hospital Organization Nagoya Medical Center Nagoya Japan; ^8^ Department of Neuropsychiatry Osaka Medical and Pharmaceutical University, Faculty of Medicine Takatsuki Japan; ^9^ Department of Psychiatry & Behavioral Science Juntendo University Graduate School of Medicine Tokyo Japan

Recent findings from genetic correlation analyses clarify the complicated relationships regarding shared genetic risk among psychiatric disorders. Using the datasets of the Psychiatric Genomics Consortium [PGC; mainly consisting of European (EUR) samples],^1^, ^2^ genetic correlations (r_g_) among bipolar disorder (BD) subtypes I/II (BD1/BD2), schizophrenia (SCZ) and major depressive disorder (MDD) revealed (1) the highest r_g_ between BD1 and BD2 (r_g_ = 0.85), (2) a higher r_g_ between BD1 and SCZ (r_g_ = 0.66) than that between BD2 and SCZ (r_g_ = 0.54) and (3) a lower r_g_ between BD1 and MDD (r_g_ = 0.34) than that between BD2 and MDD (r_g_ = 0.66).^1^

To replicate these findings, we aimed to extend the genetic relationships between BD subtypes and SCZ or MDD in East Asians (EAS): we calculated r_g_ based on Linkage Disequilibrium score regression analysis^3^ using genome‐wide association study data for BD1/2,^4^ SCZ,^5^ and MDD^6^ from EAS (Japan and China: Supplementary Text). To compare the trends in EAS, we referred to the EUR results by PGC.^1^, ^2^

We found a high genetic correlation between BD1 and BD2 in EAS, with a lower magnitude than the finding from PGC.^1^ However, different relationships were observed in EAS between BD1 and SCZ/MDD and between BD2 and SCZ/MDD (Figure 1 /Table S1). Specifically, we observed (1) a higher r_g_ between BD1 and MDD in EAS (‘mood’ spectrum), compared with the PGC results,^1^ where BD1 was closer to SCZ (“psychosis” spectrum), (2) a lower r_g_ between BD1 and SCZ in EAS, but (3) confirmed similar trends of r_g_ between BD2 and SCZ/MDD.

**Fig. 1:**
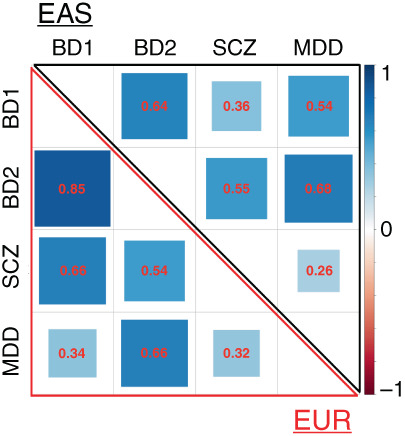
Genetic correlation between subtypes of bipolar disorder (BD1/BD2) and schizophrenia/major depressive disorder (SCZ/MDD) in East Asian and European populations. In the correlation matrix, the upper (black) and lower (red) triangles represent East Asian and European samples, respectively. The numbers correspond to scores of genetic correlation (r_g_). The European sample results are from Mullins *et al*. (Nat Genet 2021) and Howard *et al*. (Nat Neurosci 2019: for SCZ *vs*. MDD). Each difference in r_g_ between EAS and EUR populations (e.g., r_g_ for BD1 *vs*. SCZ in EAS *vs*. that in EUR) was evaluated, and all comparisons had *P* values <2 × 10^−16^. EAS: East Asian, EUR: European, SCZ: schizophrenia, BD1: bipolar disorder I, BD2: bipolar disorder II, MDD: major depressive disorder.

If the genetic risk of psychiatric disorders follows the common disease common variant (CD‐CV) hypothesis, the genetic components are shared across populations; however, we detected a discrepancy in genetic correlations between BD1/BD2 and SCZ/MDD among different ethnic populations. The current results may correspond to the discrepant trend in the prevalence of BD and SCZ. Previous epidemiological surveys reported a much lower prevalence of BD1 in EAS (~0.1%)^7^ compared with that in EUR (~1%),^7^ although BD has often been underdiagnosed and/or underreported. However, unlike BD, the prevalence of SCZ has been reported at approximately 1% worldwide, regardless of EAS and EUR.

According to our results, as well as to the CD‐CV hypothesis and epidemiological evidence, we reconsidered the diagnostic attitude towards BD1, specifically the boundaries between ‘mania with psychotic features’ (restricted to BD1 diagnosis) and other psychotic disorders; we speculate that the BD1 diagnosis in Japan adheres to ‘mood’ symptoms (e.g., mania), conversely the one in EUR is more liberal regarding ‘psychosis’ in manic phase. In this case, especially for BD1, Japanese psychiatrists may prefer to diagnose patients (and recruit them into the ‘study’) with ‘extreme’ manic symptoms ‘without’ psychotic features. Further, we assume this stance may be associated with the high genetic correlation (r_g_ = 0.54) between BD1 and MDD in EAS.

The different tendency in EUR resulted in the high r_g_ between BD1 and SCZ in the PGC (r_g_ = 0.66), since SCZ liability was enriched in BD with psychotic features (mood‐incongruent > mood‐congruent).^8^

To clarify, we surveyed the ratio of BD1 with psychotic features and found that our sample contained only approximately 30% of BD1 patients diagnosed with ‘with psychotic features’ (i.e., 177 ‘BD1 with psychotic features’ out of 631 accessible BD1 samples). In contrast, a previous study reported that half of BD1 subjects (among them approximately 2/3 ~ mood‐congruent, 1/3 ~ mood‐incongruent) in EUR exhibited psychotic features,^9^ resulting in a larger difference in the ‘psychotic features’ ratio between EAS and EUR compared to the difference of BD1 prevalence (the estimated prevalence of ‘BD1 with psychotic features’ in Japan: 0.1% × 0.3≂0.03%, that in EUR: 1% × 0.5≂0.5%). Of course, these differences may be explained by a ‘simple’ sampling bias of our datasets; however, we must be aware of the possibility that ‘diverse’ ascertainment bias (due to different diagnostic trends in different cultures) may have influenced the sample constitution in the genetic study and, moreover, possibly in clinical studies.

Nevertheless, it must be emphasized that either attitude (towards ‘mood’ or ‘psychosis’) is not always wrong; the BD1 datasets from Japan (and possibly EAS) comprise samples diagnosed with emphasis on ‘mood’ symptoms, which is appropriate for the aim of ‘dissecting’ BD1 *vs*. SCZ (typical ‘psychosis’). Conversely, the diagnosis of BD1 in Western countries potentially achieves capturing a wide range of BD patients, because they ‘straightforwardly’ followed the DSM/ICD criteria. Therefore, these results suggest that symptomatology‐based dissection is key to define novel diagnostic architecture that could standardize the diagnosis of BD1/BD2 and SCZ.^10^

In conclusion, we detected different genetic correlations among psychiatric disorders across populations. The diagnostic attitude may explain these findings, although further analysis is required to evaluate other possibilities.

## Disclosure statement

Dr. T Kanazawa received research support from Eisai, Sumitomo and Otsuka. Dr. T Kato has received research support or speaker's honoraria from the Japan Agency for Medical Research and Development (AMED), MEXT, Sumitomo Pharma, Otsuka Pharmaceutical, Takeda Pharmaceutical Co., Ltd., Eisai Co., Ltd. and Teijin Pharma; consulting fees from Sumitomo Pharma and Janssen Pharmaceutical K.K.; speaker's honoraria from Sumitomo Pharma, Otsuka Pharmaceutical, Taisho Pharma Co., Ltd., Meiji Seika Pharma, Mochida Pharmaceutical Co., Ltd., Shionogi & Co., Ltd., Janssen Pharmaceutical K.K., Janssen Asia Pacific, Yoshitomiyakuhin, MSD K.K., Kyowa Pharmaceutical Industry Co., Ltd., Takeda Pharmaceutical Co., Ltd., Taisho Pharmaceutical Co., Ltd., Eisai Co., Ltd., Viatris and Mylan N.V. and is in leadership positions in Psychiatry and Clinical Neurosciences (Editor in Chief), Brain Science Working Group of MEXT (Chair), Japanese Society of Biological Psychiatry (president), Bipolar Disorder Committee, Japanese Society for Mood Disorders (Chair) and Japanese Society for Neuroscience (Vice President). Dr. T Kishi has received speaker's honoraria from Sumitomo, Otsuka, Takeda, Eisai, Janssen and Meiji. Dr. N Iwata received research support or speakers' honoraria from Sumitomo, Eisai, Daiichi Sankyo, Takeda, Meiji, Tanabe‐Mitsubishi, Otsuka, Eli Lilly, Janssen and Viatris. The remaining authors declare no conflicts of interest.

## Supporting information


**Table S1** Genetic correlations between subtypes of bipolar disorder (BD1/BD2) and schizophrenia/major depressive disorder (SCZ/MDD) in East Asian and European populations.
