# Supplementary material for: Differential genetic correlations across major psychiatric disorders between Eastern and Western countries
Source: Psychiatry Clin Neurosci. 2022 Nov 17;77(2):118–9. doi: 10.1111/pcn.13498 (PMC10099712; doi:10.1111/pcn.13498)
Supplement: Supplementary file 1 — Table S1 Genetic correlations between subtypes of bipolar disorder (BD1/BD2) and schizophrenia/major depressive disorder (SCZ/MDD) in East Asian and European populations. [file PCN-77-118-s001.docx]

**Supplementary text:**

**Ethical statement**

For the BD and SCZ GWASs in the Japanese population^1,2^, written informed consent was obtained from all subjects following thorough explanation of the study with their anonymity preserved. The study was approved by the ethics committees of Fujita Health University and other participating universities, which are conformed to the provisions of the Declaration of Helsinki.

**Samples used in linkage disequilibrium score regression (LDSC) analysis**

For the bipolar disorder (BD) and schizophrenia (SCZ) datasets, ^1, 2^ the shared control was used (the ‘control’ subjects in SCZ Genome-wide association study (GWAS) ^2^ were completely identical to Phase1 in BD GWAS^1^: n = 7,408). In the current analysis, we randomly separated the control samples for BD (Phase 2 in the BD GWAS^1^: n = 54,291) to be completely independent: SCZ/controls = 1,941/7,408, BD I disorder (BD1)/controls = 1480/27,146 and BD II disorder (BD2)/controls = 1,380/27,145. For the major depressive disorder (MDD) GWAS of the East Asian population (EAS), we used the CONVERGE Consortium dataset comprising only Chinese females.^3^

Further, for the European (EUR) samples, we referred to the results for r_g_ for which the authors used GWAS of EUR descent collected at multiple sites.^4, 5^

**LDSC analysis**

All genetic correlation analyses were conducted using LDSC software (<https://github.com/bulik/ldsc>)^6^ to estimate the genetic correlation between two phenotypes according to the summary statistics. For quality control, we used the SNPs as follows: (1) involved in the 1000 Genomes Project and HapMap Projects, (2) minor allele frequency  ≥ 1% and other default settings of LDSC.

**Limitations**

To interpret our results, we note several limitations.

First, we were unable to conduct a genetic correlation analysis for BD1, stratified with and without psychosis in EAS, because of the limited sample size. Such an analysis is critically important to assess the validity of our hypothesis, and we; therefore, must expand the sample size and scope of the analysis.

Second, our BD samples from EAS were exclusively collected in Japan. If we can evaluate the genetic correlation between BD (and BD1/BD2) and other psychiatric disorders and check the sample constitution for BD1 between with and without psychotic features in China, these analyses assess the ‘cultural’ diagnostic trends that may differ between Japan and China and between EAS and EUR or both. Unfortunately, the GWAS results of BD for the Chinese population are not available, but the prevalence of BD and its sub-diagnosis in China are similar to those for Japan.^6^ Therefore, we assume it likely that the diagnostic attitude for BD (and possibly BD1) by Chinese psychiatrists tends to resemble that of Japanese psychiatrists.

Third, MDD for EAS was based on GWAS data collected in China, possibly creating a problem associated with the different diagnostic attitudes regarding MDD between Japan and China. Therefore, a GWAS of MDD from Japan will clarify the unique characteristic of BD1 in Japan.

Fourth, we are unable to clearly explain why the genetic correlation between BD2 and SCZ in EAS was higher compared with that between BD1 and SCZ in EAS. Indeed, the diagnostic trend for ‘psychosis’ in the manic phase is not the reason because psychiatrists in Japan (and psychiatrists in EUR) strictly follow the DSM/ICD criteria for BD2, where the diagnosis of BD2 is assigned to patients without psychotic features in manic phase (patients with psychotic features in manic phase should be diagnosed as BD1). However, based on the cross-population comparison with BD2, we note that similar magnitudes of the values of r_g_ between BD2 and SCZ/MDD were observed in each population (BD and SCZ: r_g_ = 0.55/0.54 for EAS/EUR. BD and MDD: r_g_ = 0.68/0.66 for EAS/EUR); this may indicate the similar ‘diagnostic attitude’, shared genetic aetiology, or both, for these conditions. Therefore, we speculate that the psychiatrists in EAS and EUR diagnose patients with similar ‘category of symptoms’ (‘mood’ and ‘psychosis’ in depressive phase) as BD2, introducing different situations from the case of BD1. In this assumption, such ‘similar’ magnitude for the genetic correlation between BD2 and SCZ/MDD in each population might not be associated with different prevalence of BD2 between EAS and EUR (*vice versa*): the lower prevalence of BD2 in EAS (BD1/BD2: 0.1/0.1% in Japan,^7^ 0.09/0.04% in China^8^) compared with that in EUR (1.0/1.1% in US,^7^ 1.0/0.7% in New Zealand^7^). If so, again, we do not have any clear explanation for the different prevalence of BD2 between EAS and EUR except that BD could have been underdiagnosed or underreported in the clinical setting or epidemiological surveys reported previously.^9^ In any case, we assume these findings convincingly support our conclusion that BD1 in Japan is associated with a unique diagnostic feature, but ascertainment bias is minimal for BD2.

**Author Contributions:**

TS, MI, TKanazawa, TKato and NI contributed to the conception and study design. TS, MI, CT, TA, ST, MM and TKishi provide substantial contributions to analysis and interpretation of clinical data. TS, MI and CT wrote the first draft of the article. All authors have contributed to and approved the final version of the manuscript.

**References**

1. Ikeda M, Takahashi A, Kamatani Y, et al. A genome-wide association study identifies two novel susceptibility loci and trans population polygenicity associated with bipolar disorder. *Mol Psychiatry*. 2018; **23**: 639-47.

2. Ikeda M, Takahashi A, Kamatani Y, et al. Genome-Wide Association Study Detected Novel Susceptibility Genes for Schizophrenia and Shared Trans-Populations/Diseases Genetic Effect. *Schizophr Bull*. 2019; **45**: 824-34.

3. Converge Consortium. Sparse whole-genome sequencing identifies two loci for major depressive disorder. *Nature*. 2015; **523**: 588-91.

4. Mullins N, Forstner AJ, O'Connell KS, et al. Genome-wide association study of more than 40,000 bipolar disorder cases provides new insights into the underlying biology. *Nat Genet*. 2021; **53**: 817-29.

5. Howard DM, Adams MJ, Clarke TK, et al. Genome-wide meta-analysis of depression identifies 102 independent variants and highlights the importance of the prefrontal brain regions. *Nat Neurosci*. 2019; **22**: 343-52.

6. Bulik-Sullivan B, Finucane HK, Anttila V, et al. An atlas of genetic correlations across human diseases and traits. *Nat Genet*. 2015; **47**: 1236-41.

7. Merikangas KR, Jin R, He JP, et al. Prevalence and correlates of bipolar spectrum disorder in the world mental health survey initiative. *Arch Gen Psychiatry*. 2011; **68**: 241-51.

8. Zhang L, Cao XL, Wang SB, et al. The prevalence of bipolar disorder in China: A meta-analysis. *J Affect Disord*. 2017; **207**: 413-21.

9. Nishi D, Ishikawa H, Kawakami N. Prevalence of mental disorders and mental health service use in Japan. *Psychiatry Clin Neurosci*. 2019; **73**: 458-65.

| population | phenotype1 | phenotype2 | r_g_ | Standard error | P-value |
| --- | --- | --- | --- | --- | --- |
| East Asian | BD | SCZ | 0.460 | 0.0879 | 1.70E−07 |
| (EAS) |  | MDD | 0.675 | 0.118 | 1.16E−08 |
|  | BD1 | BD2 | 0.641 | 0.224 | 0.00420 |
|  |  | SCZ | 0.357 | 0.0896 | 6.73E−05 |
|  |  | MDD | 0.539 | 0.126 | 1.98E−05 |
|  | BD2 | SCZ | 0.552 | 0.188 | 0.00320 |
|  |  | MDD | 0.681 | 0.209 | 0.00110 |
|  | SCZ | MDD | 0.262 | 0.110 | 0.0169 |
| European | BD | SCZ | 0.685 | 0.0172 | 0 |
| (EUR) |  | MDD | 0.441 | 0.0245 | 2.17E−72 |
|  | BD1 | BD2 | 0.846 | 0.0545 | 2.88E−54 |
|  |  | SCZ | 0.660 | 0.0192 | 2.10E−258 |
|  |  | MDD | 0.339 | 0.0264 | 7.83E−38 |
|  | BD2 | SCZ | 0.544 | 0.0465 | 1.10E−31 |
|  |  | MDD | 0.656 | 0.0513 | 1.95E−37 |
|  | SCZ | MDD | 0.323 | 0.0235 | 3.55E−43 |

**Supplementary Table 1**: Genetic correlations between subtypes of bipolar disorder (BD1/BD2) and schizophrenia/major depressive disorder (SCZ/MDD) in East Asian and European populations.

SCZ: schizophrenia, BD: bipolar disorder, MDD: major depressive disorder.

The European sample results are from Mullins et al. (Nat Genet 2021) and Howard et al. (Nat Neurosci 2019: for SCZ vs. MDD).
